# Antigen specificity affects analysis of natural antibodies

**DOI:** 10.3389/fimmu.2024.1448320

**Published:** 2024-08-07

**Authors:** Kendra Weston, Janet E. Fulton, Jeb Owen

**Affiliations:** ^1^ Department of Entomology, Washington State University, Pullman, WA, United States; ^2^ Hy-Line International, Dallas Center, IA, United States

**Keywords:** chickens, disease ecology, immunoglobulin, innate immunity, selection

## Abstract

Natural antibodies are used to compare immune systems across taxa, to study wildlife disease ecology, and as selection markers in livestock breeding. These immunoglobulins are present prior to immune stimulation. They are described as having low antigen specificity or polyreactive binding and are measured by binding to self-antigens or novel exogenous proteins. Most studies use only one or two antigens to measure natural antibodies and ignore potential effects of antigen specificity in analyses. It remains unclear how different antigen-specific natural antibodies are related or how diversity among natural antibodies may affect analyses of these immunoglobulins. Using genetically distinct lines of chickens as a model system, we tested the hypotheses that (1) antigen-specific natural antibodies are independent of each other and (2) antigen specificity affects the comparison of natural antibodies among animals. We used blood cell agglutination and enzyme-linked immunosorbent assays to measure levels of natural antibodies binding to four antigens: (i) rabbit erythrocytes, (ii) keyhole limpet hemocyanin, (iii) phytohemagglutinin, or (iv) ovalbumin. We observed that levels of antigen specific natural antibodies were not correlated. There were significant differences in levels of natural antibodies among lines of chickens, indicating genetic variation for natural antibody production. However, line distinctions were not consistent among antigen specific natural antibodies. These data show that natural antibodies are a pool of relatively distinct immunoglobulins, and that antigen specificity may affect interpretation of natural antibody function and comparative immunology.

## 1 Introduction

Natural antibodies (NAbs) are a class of immunoglobulins present in blood for which the immune system has had no prior stimulation (1–5) and which can function in the innate immune response (6–8). These immunoglobulins are variously described as polyreactive or having low antigen specificity (8–10). Natural antibodies are commonly measured using antigens that animals have not previously encountered such as keyhole limpet hemocyanin (KLH) or self-antigens like ovalbumin (OVA) (11–13). The NAbs may be directly protective by neutralizing pathogens (14–16), and they may facilitate the adaptive immune response by supporting pathogen phagocytosis and antigen presentation (11, 17). Due to their defensive properties and their function as a bridge between the innate and adaptive compartments of the immune system, NAbs have been studied in the contexts of disease, ecology, evolution, and comparative immunology (15, 18–20). Experimental infections under laboratory conditions have shown that higher levels of NAbs are associated with lower infection intensities (21) and a higher probability of survival (1, 22). However, some NAbs appear inversely associated with higher survival probability (22). Studies of wildlife suggest that individuals with higher levels of NAbs have greater bactericidal capabilities (8, 18, 23, 24) and resistance to parasites (7, 25, 26). The NAbs appear to be correlated with other immunological factors (27–29) and vary among sexes (30), populations (31), life stages (32), food resources (33–35), and seasons (36). In animal agriculture, NAbs have been used as selection traits to enhance disease resistance (37–42). Finally, given that NAbs are thought to bridge innate and adaptive immune responses, they have been used to compare immune systems among different taxa (27, 28, 43, 44) and to explore tradeoffs in the evolution of immune systems (19, 30). Other than the mouse (*Mus musculus*), the chicken (*Gallus gallus*) is the most widely used animal model for the study of NAbs (1, 3, 6, 22, 27, 33, 39–42, 45–61). As early as 1923 natural antibodies were identified in chickens (53). Subsequently, NAbs have been linked to poultry survival (1, 45, 56) and pathogen resistance (39–41). The genetic basis for variation in NAbs has been explored using established genetic lines of poultry (6, 22, 40, 42, 47–49, 52, 55) and with modern genomics tools (62–66). The chicken has been a vital model for developing NAb assays used for wild bird species (25, 27, 28, 43). However, given the agricultural and economic importance of poultry, the primary application of NAb research on chickens has been in selective breeding for enhanced immune defense (39–42, 46, 49, 51, 58, 60).

Although some functional studies of NAbs are based on isolated B-1 cell lines in mice (18, 67–71), most studies of NAbs are based on measures of circulating immunoglobulins in serum or plasma (6, 18, 37, 38, 43). These measures are commonly done using one of two methods – (1) hemagglutination assay (HA) or (2) enzyme-linked immunosorbent assay (ELISA). Hemagglutination assays measure *in vitro* antibody binding and cross-linking of vertebrate red blood cells (72). In hemagglutination assays, antibodies bind to proteins on the surface of red blood cells, causing clumping (agglutination) of the red blood cells in a microtiter well (17, 72). The antibody titer is calculated based on a dilution series of sera or plasma incubated with a fixed concentration of red blood cells, and the experimenter determines the dilution point when agglutination fails to occur (3, 27). Hemagglutination assays are often employed in wildlife studies (23, 28, 30, 44) and are popular because they do not require special reagents or equipment (27). The ELISA uses a microtiter plate coated with a specific antigen and then filled and incubated with sera or plasma from the study animal. Antibodies in the sera/plasma that bind to the plate-affixed antigen can be quantified using a secondary antibody conjugated to a colorimetric enzyme or fluorescence marker detected by a spectrophotometer (17, 72, 73). The ELISA approach is frequently used in laboratory and livestock research (43, 74–76). Different antigens are used to measure NAbs with both methods. Hemagglutination assays may use blood cells from rabbits, sheep, chickens, or fish (27, 47–50, 77). Enzyme-linked immunosorbent assays may use antigens from bacteria (e.g., lipopolysaccharide), invertebrates (e.g., keyhole limpet hemocyanin), plants (e.g., phytohemagglutinin), or vertebrates (e.g., ovalbumin) (11, 51, 52).

These different methods measure NAbs binding to specific antigens, and it is unclear how these antigen-specific NAbs are related. Though NAbs are expected to be polyreactive (24, 78–81), studies of monoclonal NAbs reveal that NAbs do not bind equally well to all antigens. For example, Gunti et al. (8) showed that NAbs from a single B-1 clone exhibited wide variation in binding to different bacteria, including species from the same genus. Importantly, different NAbs can have unique (non-overlapping) ranges of antigen binding (82, 83). Baumgarth et al. (9, 84) argue that immunological defense by NAbs results from broad antigen reactivity of both individual NAbs and the natural antibody repertoire. These studies underscore that NAbs are not uniform and the NAb repertoire reflects a diverse pool of immunoglobulins with potentially unique but complimentary antigen specificities. Despite these insights, the majority of NAb studies are based on immunoglobulin measures using only one or two antigens (6, 19, 22, 27, 28, 30, 34, 42, 49, 51, 60, 74, 76, 85–97). This raises an important question – are NAbs measured with one antigen representative of the broader repertoire of NAbs? This is a vital question, given that many studies of NAbs infer immune function (14, 22, 27, 30, 76, 86, 91, 98), ecological relationships (25, 28, 34, 99), and evolution (19, 25, 42, 49, 97, 100) based on one or two NAb antigens. Additionally, the antigens used vary widely across studies (22, 27, 60, 89), which makes it difficult to synthesize results among different study systems.

To our knowledge, comparisons of circulating NAbs have not been reported in the literature. Here, we tested two related hypotheses regarding NAb specificity (1): Levels of antigen-specific NAbs are independent; (2) Antigen specificity affects comparison of natural antibodies among animals. We tested these hypotheses by measuring NAb levels among genetically distinct chicken breeds and selection lines, using different antigens with agglutination and ELISA methods. We discuss the potential importance of antigen specificity to the study of NAb function and comparative immunology.

## 2 Materials and methods

### 2.1 Poultry

Plasma was collected from female chickens in eight selection lines across three breeds: White Leghorn (WL), White Plymouth Rock (WPR), and Rhode Island Red (RIR). These three breeds are distinct, having been historically selected for different physical characteristics including feather color and eggshell color. Each was defined as a different breed over 100 years ago (101). The different lines within each breed have been separated from one another since the 1940s through artificial selection on egg production, body weight, and feed conversion efficiency (102). The Rhode Island Red breed is a cross of three breeds (Malay Game, Leghorn, and Asian native) originating in Europe around 100 B.C (103, 104). The Leghorn breed originated in Italy in 400 B.C. and are classified as egg layers (103, 104) and are the most commonly utilized breed for white egg shell production. They exhibit a range of plumage colors, with white being the most common (104). The White Plymouth Rock was one of the breeds used to develop the modern commercial meat (broiler) bird in the early part of the 20^th^ century (104). The Rhode Island Red and Plymouth Rock breeds are used for commercial brown egg production. All birds used in this study were from elite egg layer lines, selected for generations for multiple egg production related traits. These are all pure lines, not commercial cross production birds. They were maintained at the breeding facilities of Hy-Line International. The housing was in single cages, under standard production conditions with feed and water provided *ad libitum*.

### 2.2 Plasma samples

Blood was collected from birds in each line at ten weeks of age, and plasma samples were separated from blood by centrifugation at 240 x g for three minutes. After collection, plasma samples were split into aliquots for the different assays, then stored at -20°C until use. Sample sizes ranged from 98–106 birds per line with eight lines total across three breeds (two for Rhode Island Red, four for White Leghorn, and two for White Plymouth Rock), with a total of 813 samples for the ELISA assays, and sample sizes ranged from 10–25 birds per line, with a total of 119 samples for the rabbit blood cell hemagglutination (HA) assay.

### 2.3 Agglutination assay

Measures of NAbs binding to rabbit red blood cells (anti-rRBC NAbs) were determined using the hemagglutination assay (HA) (27, 28). A 1% rabbit blood cell (RBC) suspension was prepared using rabbit whole blood in Alsever’s solution (catalog # RBA050, Hemostat Laboratories, Dixon, CA, USA). Briefly, RBCs in whole blood were washed four times with centrifugation at 241.5 x g for 5 min in phosphate-buffered saline (PBS, catalog# P3813, Sigma-Aldrich, St. Louis, MO, USA). Lysed red blood cells were removed with the supernatant after each wash. The washed cell concentration was measured using duplicate hematocrit capillary tubes following sample centrifugation at 0.004 x g for 1 min. Additional PBS was added to make a 1% red blood cell suspension with confirmation of the concentration via hematocrit. Each HA assay was done using a 96-well microtiter plate (CorningTM Clear Polystyrene 96-Well Microplates, catalog# 3795, Thermo Scientific, Rockford, IL, USA). Each row of wells tested plasma from a single bird, with 2x dilutions of the plasma from the first well (25 µl undiluted plasma) through the 11^th^ well (1/1024 dilution of plasma in PBS; 25 µl total volume). Seven rows contained experimental samples (7 birds) and one row contained a positive control sample of pooled plasma. Column 12 wells contained 25µl PBS only, as a negative control. After plasma samples were distributed, 25µl of the 1% rabbit blood cell suspension were added to each well. The plates were sealed with Parafilm to prevent evaporation. Plates were placed on an orbital for 10 seconds and incubated in a 37°C water bath for 90 min. Plates were removed and placed at a 45-degree angle (on the long axis) for 20 min at room temperature to enhance the visualization of agglutination. We photographed and re-sealed each plate before incubation for an additional 70 minutes at a 45-degree angle at room temperature. We then photographed each plate again. Chicken samples were numerically coded and randomized across plates to avoid observer bias. The plates were scored blindly at the time of assay. The same researcher scored the photos of assays later to validate the original scores (27). Scores were recorded as the highest dilution (i.e., well number) at which agglutination occurred. Due to time constraints, we did not conduct HA assays on all plasma samples. A random subset of samples from each line of chicken breed was selected. The HA sample sizes matched or exceeded sample sizes reported in the literature for this assay (27, 28, 105–107). Sample sizes for the HA assay were Rhode Island Red (n=29), White Leghorn (n=59), and White Plymouth Rock (n=31) (**Table 1**).

**Table 1:** Sample sizes (n) of three breeds (Rhode Island Red, White Leghorn, and White Plymouth Rock) consisting of eight lines (RIR 1, RIR 2, WL 1, WL 2, WL 3, WL 4, WPR 1, and WPR 2) for assays (ELISA and HA) used to measure natural antibodies.

	Breed	n	Line	n
ELISA	*Rhode Island Red*	203	RIR 1	102
			RIR 2	101
	*White Leghorn*	402	WL 1	100
			WL 2	100
			WL 3	100
			WL 4	102
	*White Plymouth Rock*	208	WPR 1	106
			WPR 2	102
HA	*Rhode Island Red*	29	RIR 1	16
			RIR 2	13
	*White Leghorn*	59	WL 1	10
			WL 2	18
			WL 3	18
			WL 4	13
	*White Plymouth Rock*	31	WPR 1	14
			WPR 2	17

### 2.4 Enzyme-Linked Immunosorbent Assay (ELISA)

We developed indirect ELISAs to quantify IgY NAbs binding to each of three antigens - keyhole limpet hemocyanin (KLH; Hemocyanin from *Megathura crenulate* (keyhole limpet), catalog # H7017–20MG, Sigma-Aldrich, St. Louis, MO, USA), phytohemagglutinin (PHA; Phytohemagglutinin-L, catalog # 11249738001, Sigma-Aldrich, St. Louis, MO, USA), and ovalbumin (OVA; Imject ™ Ovalbumin, catalog # 77120, Thermo Scientific, Rockford, IL, USA) using a single dilution approach (108–110). Each assay was optimized based on dilutions of antigen and plasma (i.e., checkerboard titration) with the following steps: (Step 1) 96-well clear microtiter plates (Immulon ^®^ 4HBX Flat Bottom Microtiter^®^ Plates, Thermo Scientific, Rockford, IL, USA) were coated with an antigen diluted in *coating buffer* (Carbonate-Bicarbonate Buffer, catalog # C3041–100CAP, Sigma-Aldrich, St. Louis, MO, USA), with 100µl of solution per well. Antigen concentrations ranged from 1x (stock solution) in row A to 1:2000 dilution in row H. The plate was incubated at room temperature on an orbital shaker for 1 hour. (Step 2) The plate was washed with *Tris buffer* (Tris Buffered Saline, with Tween ^®^ 20, pH 8.0, catalog # T9039–10PAK, Sigma-Aldrich, St. Louis, MO, USA) three times. (Step 3) The wells were filled (300 µl/well) with *blocking buffer* (Pierce ™ Protein-Free Blocking Buffer, catalog # 37572, Thermo Scientific, Rockford, IL, USA) to cover any open binding surfaces on the plate. The plate was incubated at room temperature on an orbital shaker for 30 minutes. (Step 4) The plate was washed with *Tris buffer* three times. (Step 5) A pooled plasma sample was diluted in *sample buffer* (50 mM Tris buffered saline, pH 8.0, 1% BSA; Sigma Chemical # T6789; 10% Tween 20, Tween 20; Sigma Chemical P7949) and added to the plate (100µl/well) so that plasma concentrations ranged from 1x (undiluted) in column one to 1:2048 in column 12. The plate was incubated at room temperature on an orbital shaker for 1 hour. (Step 5) The plate was washed with *Tris buffer* three times. (Step 6) D*etection antibody* (anti-Chicken IgY – Fc Fragment Antibody with HRP conjugate; A30–104P, Bethyl Laboratories Inc., Texas, USA) was diluted in *sample buffer* 1:200,000 and added to all wells (100µl/well). The plate was covered with aluminum foil and incubated on an orbital shaker for 1 hour at room temperature. (Step 7) The plate was washed with *Tris buffer* three times. (Step 8) 100µl TMB (3,3’,5,5’-Tetramethylbenzidine Liquid Substrate System, catalog # T8665–1L, Sigma-Aldrich, St. Louis, MO, USA) were added to each well and the plate was incubated on an orbital shaker for 15 min. (Step 9) We added 100µl of *stop solution* (BioFX ^®^ 450nm Liquid Nova-Stop Solution for TMB Microwell Substrates, NC1026538, Thermo Scientific, Rockford, IL, USA) to each well. (Step 10) The plate was scanned at 450 nm with a spectrophotometer (Imark™ Microplate reader, Bio-RAD, Hercules, CA, USA) to determine the optical density (OD) in each well. Optical density correlates with the amount of antibody bound to the substrate antigen. We confirmed that OD values decreased as antigen dilution increased and as plasma dilution increased. This validated antibody-antigen binding in the assay. We compared the OD curves for the plasma dilutions among the different antigen concentrations. We selected the antigen concentration that yielded an OD curve with the steepest slope across plasma dilutions, because this reflected the antigen concentration most sensitive to changes in antibody concentration. Once the optimal concentration was determined for each antigen, the selected concentration was used for all sample assays. The selected antigen concentrations were KLH (1:40 dilution), PHA (1:500 dilution), and OVA (1:20 dilution). All assays used plasma samples diluted 1:100 and each sample was analyzed in triplicate wells (100µl/well). In addition, all assays included a positive control sample of pooled plasma and a negative control of *sample buffer* only. The OD value of the negative control (buffer only) was subtracted from all sample wells, and we determined the coefficient of variation (CV) for the optical densities of triplicate wells for each sample. For samples with a CV <20%, we averaged the triplicate well OD values to use in statistical analyses. Samples with CV values >20% were excluded from analyses. Sample sizes for ELISAs were as follows: Rhode Island Red (n=203), White Leghorn (n=402), and White Plymouth Rock (n=208) (**Table 1**).

### 2.5 Statistical methods

All sample data were standardized against positive controls by calculating the ratio of the sample value (S_i_) to the value of the positive control (P) on the plate (*x_i_ =* S_i_/P). This controlled any assay variation among different plates. We then normalized S/P values (*x_i_*) of each assay by scaling from 0 to 1 with the following formula: *z_i_* = (*x_i_* – min(x))/(max(x) - min(x)), where *x* = (*x*_1_,…,*x_n_*) and *z_i_* is the *i^th^* normalized data value. This was done to enable direct comparisons of assay results on a common scale. To analyze relationships between antigen-specific NAbs, we calculated Spearman’s correlation coefficients for each pairwise combination of NAb assay antigens with the *psych* package in R (111). We expected that any correlated NAbs would reflect cross-reactivity of polyreactive NAbs or tightly linked production of antigen-specific NAbs. Conversely, uncorrelated NAbs would indicate antigen-specific NAbs are independent. We compared NAb levels among chicken breeds and lines for each antigen-specific NAb using Generalized Linear Models (GLM) with *stats package* in R (112). The GLM models were NAb_x_ level ~ Line + Breed, where NAb_x_ represents the normalized assay values for antigen x. *Post hoc* pairwise comparisons were evaluated using the *emmeans package* (113). Significant effects of chicken line or breed on NAb levels indicate genetic variation for NAb expression. Statistical differences in NAb levels among lines and breeds were qualitatively compared among assay antigens, to determine if antigen specificity affects comparisons of NAbs among genetically distinct animals.

## 3 Results

### 3.1 Correlations among antigen-specific NAbs

**Figure 1:**
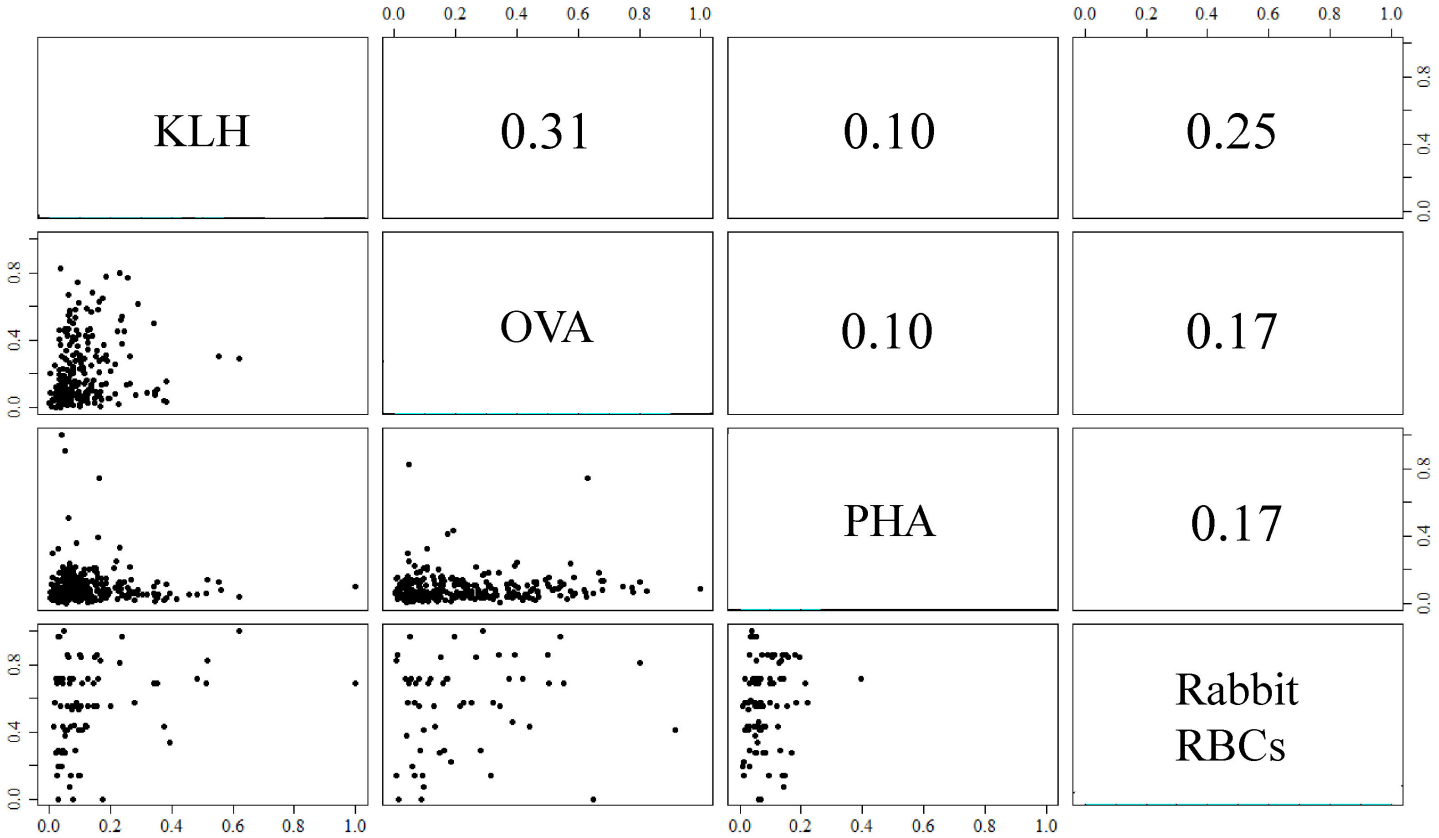
Pairwise correlations among levels of antigen-specific natural antibodies (NAbs) binding molecules ovalbumin (OVA), keyhole limpet hemocyanin (KLH), phytohemagglutinin (PHA), and rabbit red blood cells (RBCs). The NAb measures were normalized to a 0 to 1 scale for comparison. Antigen-specific NAb expression of individual birds is shown in scatter plots below the diagonal and Spearman’s correlation coefficients of NAb expression above the diagonal.

The correlation coefficients of antigen-specific NAb levels ranged from 0.10 to 0.31, indicating that antigen-specific NAb levels were independent (**Figure 1**). As a result, antigen-specific NAbs were considered independent and unique subsets of the overall NAb repertoire.

### 3.2 Agglutination assays of NAbs among breeds and lines

**Figure 2:**
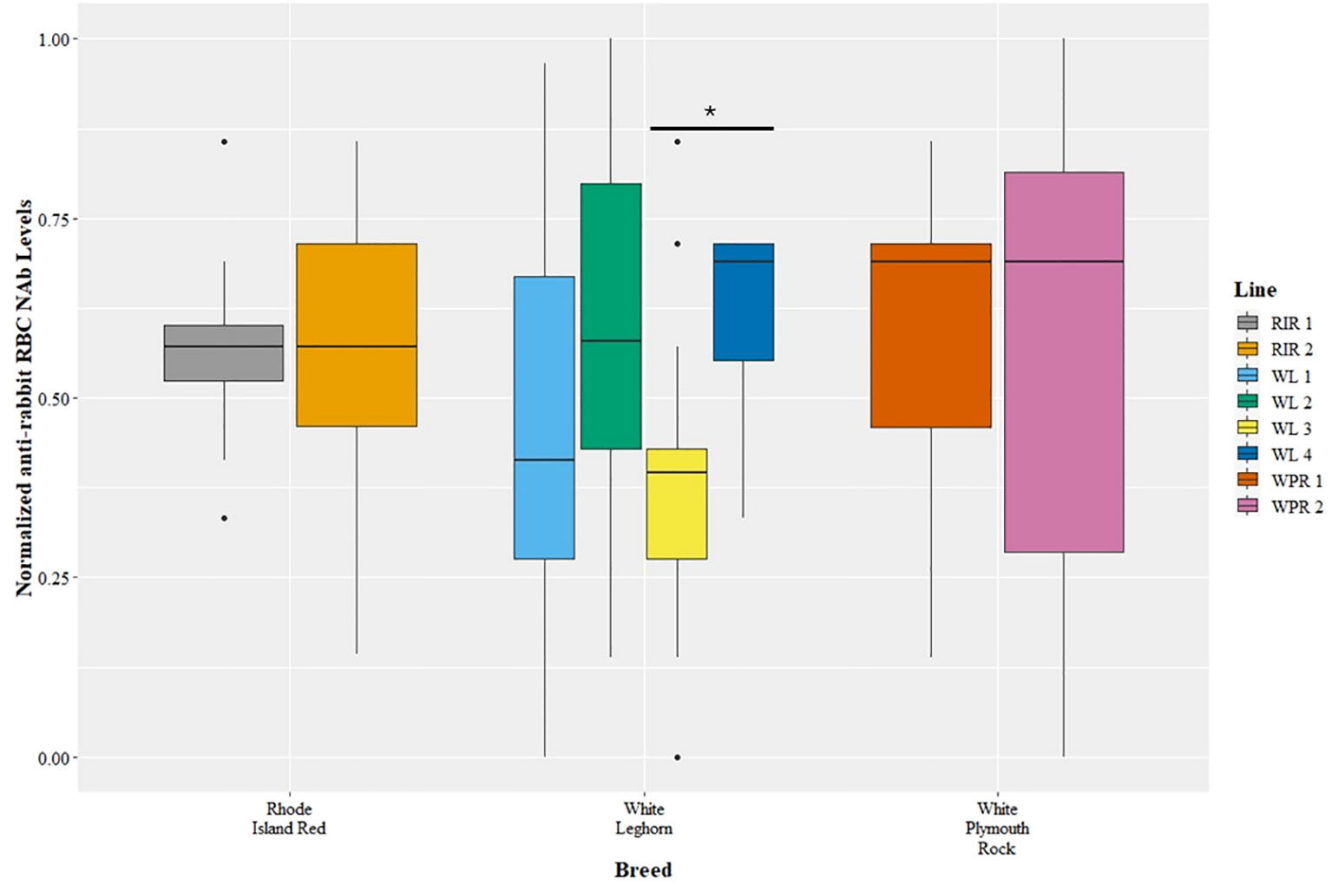
Levels of natural antibodies (NAb) binding rabbit red blood cells (rRBCs) were measured with hemagglutination assays. The anti-rRBC NAb levels were measured as the highest dilution of plasma that resulted in RBC agglutination. The anti-rRBC NAb levels were normalized to a 0 to 1 scale before comparison among breeds and lines. The NAb measures are shown for multiple breeds (x-axis) and lines (legend), with points representing outliers. There was a significant difference in anti-rRBC NAb level between lines WL3 and WL4 (*post hoc*, *p* < 0.05), but no differences among breeds.

The genetic line of chickens affected anti-rRBC NAbs (GLM, *t*=2.56, *p*=0.01), but there was no effect of breed (GLM, *t*=1.30, *p*=0.20) (**Figure 2**). White Leghorn Line 3 had lower levels of anti-rRBC NAbs compared to line White Leghorn Line 4 (GLM, *t*=3.20, *p*=0.04), but no other pairwise line differences were observed.

### 3.3 ELISAs of NAbs among breeds and lines

**Figure 3:**
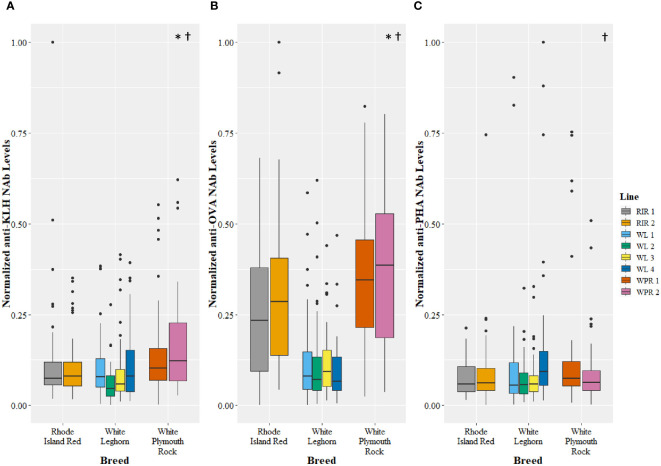
Levels of natural antibodies (NAb) binding keyhole limpet hemocyanin (KLH) **(A)**, ovalbumin (OVA) **(B)**, and phytohemagglutinin (PHA) **(C)** measured using enzyme-linked immunosorbent assays and recorded as optical density units with spectrophotometry. The optical density values were normalized to a 0 to 1 scale before comparison among breeds and lines. The antigen-specific NAb levels are shown for multiple breeds (x-axis) and lines (legend), with single points representing outliers. Symbols represent significant differences (*p*<0.05). The (*) symbol indicates a difference in NAb levels among breeds, and the (†) symbol indicates a difference in NAb levels among lines. See **Tables 2**, **3** for pairwise comparisons.

The genetic line and breed of chicken affected levels of NAbs measured in all three ELISAs (**Figure 3**). Among the poultry lines there was a difference in expression of NAbs binding KLH (GLM, *t*=3.41, *p*=0.001), OVA (GLM, *t=*4.32, *p*<0.0001), and PHA (GLM, *t*=3.80, *p*=0.0002). *Post hoc* pairwise comparisons of lines revealed significant differences between 5 pairs of lines in the KLH assay, 17 pairs of lines in the OVA assay, and 5 pairs of lines in the PHA assay (**Table 2**). Among the poultry breeds, there was a difference in the expression of NAbs binding KLH (GLM, *t*=2.27, *p*=0.02) and OVA (GLM, *t*=8.20, *p*<0.001), but no difference in the expression of NAbs binding PHA (GLM, *t*=1.55, *p*=0.12). *Post hoc* pairwise comparisons of breeds revealed significant differences between 2 pairs of lines in the KLH assay, 3 pairs of lines in the OVA assay, and no pairs of lines in the PHA assay (**Table 3**).

**Table 2:** Pairwise comparisons of NAbs binding KLH, OVA, PHA, and rRBCs among poultry lines (RIR 1, RIR 2, WL 1, WL 2, WL 3, WL 4, WPR 1, and WPR 2).

	Contrast	Estimate	SE	df	t ratio	p value
KLH	*RIR 1 - WL 2*	0.066	0.019	459	3.409	0.0162
	*WL 1 - WPR 2*	-0.063	0.020	459	-3.098	0.0428
	*WL 2 - WPR 1*	-0.081	0.018	459	-4.423	0.0003
	*WL 2 - WPR 2*	-0.106	0.020	459	-5.206	0.0000
	*WL 3 - WPR 2*	-0.078	0.020	459	-3.898	0.0028
OVA	*RIR 1 - WL 1*	0.136	0.031	370	4.318	0.0005
	*RIR 1 - WL 2*	0.141	0.034	370	4.214	0.0008
	*RIR 1 - WL 3*	0.127	0.035	370	3.567	0.0096
	*RIR 1 - WL 4*	0.154	0.037	370	4.202	0.0009
	*RIR 1 - WPR 2*	-0.113	0.034	370	-3.323	0.0217
	*RIR 2 - WL 1*	0.192	0.031	370	6.269	*p*<0.0001
	*RIR 2 - WL 2*	0.198	0.033	370	6.027	*p*<0.0001
	*RIR 2 - WL 3*	0.183	0.035	370	5.257	*p*<0.0001
	*RIR 2 - WL 4*	0.210	0.036	370	5.851	*p*<0.0001
	*WL 1 - WPR 1*	-0.233	0.030	370	-7.738	*p*<0.0001
	*WL 1 - WPR 2*	-0.249	0.031	370	-8.024	*p*<0.0001
	*WL 2 - WPR 1*	-0.238	0.032	370	-7.386	*p*<0.0001
	*WL 2 - WPR 2*	-0.254	0.033	370	-7.676	*p*<0.0001
	*WL 3 - WPR 1*	-0.224	0.034	370	-6.520	*p*<0.0001
	*WL 3 - WPR 2*	-0.240	0.035	370	-6.823	*p*<0.0001
	*WL 4 - WPR 1*	-0.251	0.035	370	-7.080	*p*<0.0001
	*WL 4 - WPR 2*	-0.267	0.036	370	-7.367	*p*<0.0001
PHA	*RIR 1 - WL 4*	-0.060	0.016	658	-3.802	0.0039
	*RIR 2 - WL 4*	-0.049	0.016	658	-3.049	0.0489
	*WL 2 - WL 4*	-0.066	0.016	658	-4.234	0.0007
	*WL 3 - WL 4*	-0.062	0.015	658	-4.027	0.0016
	*WL 4 - WPR 2*	0.052	0.015	658	3.378	0.0175
rRBC	*WL 3 - WL 4*	-0.262	0.082	111	-3.188	0.0380

*tukey p-value adjustment for multiple comparisons.

Only significant comparisons are shown (*p*<0.05).

**Table 3:** Pairwise comparisons of NAbs binding KLH, OVA, PHA, and rRBCs among poultry breeds (Rhode Island Red, White Leghorn, and White Plymouth Rock).

	Contrast	Estimate	SE	df	t ratio	p value
KLH	*Rhode Island Red - White Leghorn*	0.027	0.012	464	2.265	0.0618
	*Rhode Island Red - White Plymouth Rock*	-0.033	0.014	464	-2.364	0.0480*
	*White Leghorn - White Plymouth Rock*	-0.060	0.012	464	-5.067	*p*<0.0001*
OVA	*Rhode Island Red - White Leghorn*	0.168	0.020	375	8.201	*p*<0.0001*
	*Rhode Island Red - White Plymouth Rock*	-0.075	0.023	375	-3.215	0.0040*
	*White Leghorn - White Plymouth Rock*	-0.243	0.020	375	-12.092	*p*<0.0001*
PHA	*Rhode Island Red - White Leghorn*	-0.014	0.010	663	-1.355	0.3654
	*Rhode Island Red - White Plymouth Rock*	-0.018	0.012	663	-1.546	0.2701
	*White Leghorn - White Plymouth Rock*	-0.004	0.010	663	-0.443	0.8975
rRBC	*Rhode Island Red - White Leghorn*	0.069	0.053	116	1.300	0.3980
	*Rhode Island Red - White Plymouth Rock*	-0.019	0.061	116	-0.306	0.9497
	*White Leghorn - White Plymouth Rock*	-0.088	0.052	116	-1.686	0.2150

*tukey p-value adjustment for multiple comparisons.

Significant differences are indicated by the * symbol (*p*<0.05).

## 4 Discussion

We compared natural antibody levels among different breeds and selected elite lines of poultry using two methods (i.e. ELISA and HA) to test the hypotheses that (1) antigen-specific NAbs are independent and (2) antigen specificity affects comparisons of NAbs among different animals. Our data supported both hypotheses. Correlations among antigen-specific NAbs were absent or weak, indicating that the measured NAbs did not have strongly overlapping antigen binding (i.e., similar polyreactivity), nor were they tightly linked (i.e. co-regulated). We observed significant differences in levels of antigen-specific NAbs among chicken breeds and lines, reinforcing the concept that NAbs are germline-encoded (6, 22, 40, 42, 48, 49, 52, 55). However, breed and line effects were not consistent among antigen-specific NAbs. For example, all three breeds differed in levels of anti-KLH and anti-OVA NAbs, but not anti-rRBC nor anti-PHA NAbs. Relative differences were not consistent among antigen-specific NAbs. For example, White Leghorn chickens had lower levels of anti-OVA NAbs compared to Rhode Island Red chickens, but levels of anti-KLH NAbs were similar between the two breeds. Levels of anti-OVA NAbs were different among all eight lines. In comparison, anti-KLH and -PHA NAbs were different among six lines, and anti-rRBCs NAbs differed among only two lines. These data show that subsets of NAbs have some degree of unique (non-overlapping) antigen specificity and are not tightly linked. Genetic differences among chickens affect levels of antigen-specific NAbs, but these genetic effects (i.e., breed and line differences) were not consistent among antigen-specific NAbs. These results raise important questions about the diversity of immunoglobulins in the NAb repertoire and the potential importance of antigen-specificity to NAb function and comparative immunology.

A hallmark of NAbs is polyreactivity (8, 9, 114) and binding to diverse antigens has been clearly demonstrated with studies using NAbs from monoclonal B-1 cells (7–9, 18, 24, 26). However, evidence in the literature illustrates that NAbs can have unique, non-overlapping ranges of antigen binding (8, 24) and structural diversity at the antigen binding site (114). Several reviews of NAbs have mentioned diversity of immunoglobulins in the NAb repertoire (11, 13) and this diversity is suggested to be essential to immune defense (9, 84). Despite this information, nearly all studies of NAbs are based on one or two antigens and the antigens used among studies are often different. Should we expect that measures of antigen-specific NAbs represent the broader NAb repertoire or reflect the activity of other natural antibodies? Evidence from the literature suggests that antigen-specific NAbs are not interchangeable.

In two notable studies, different red blood cells were used for hemagglutination assays of NAbs from the same animals. Bailey (53) observed that poultry sera agglutinated rabbit and rat red blood cells at a titer four times higher than the agglutination threshold for red blood cells from guinea pigs or frogs, and >20 times higher than the threshold for cells from sheep, turtle, or goat blood (53). Seto and Henderson (3) observed poultry sera agglutinated mouse red blood cells at a titer 1.5x higher than hamster red blood cells and 12x higher than blood cells from rabbits or sheep (3). The wide variation in NAb titers among different vertebrate red blood cells indicates that each assay measured a different antigen-specific natural antibody. This is perhaps unsurprising, given more recent analyses of the protein composition of red blood cells. Using quantitative mass spectrometry, Sae-Lee et al. (115) identified 1,944 distinct protein groups in human red blood cells, and Matei et al. (116) described multiple electrophoretic differences in red blood cell proteins among eight animal species, including sheep, rabbit, rat, and mouse. Molecular analyses of antigens used in NAb ELISAs also suggest significant structural diversity that could affect binding specificity. The KLH molecule, derived from the mollusk *Megathura crenulate*, is a ~390 kDa polypeptide with eight globular units (117). The OVA molecule, derived from egg white, is a 44.5 kDa glycoprotein with a tertiary structure (118, 119). Finally, a legume plant produces the PHA molecule, a 30.5 kDa glycoprotein with a quaternary structure (120). As with the agglutination assay, these molecular differences likely contribute to epitope variation among ELISAs. These antigen and epitope differences among NAb assays likely result in measurements of different subsets of NAbs (24, 70, 78, 121) which may affect analyses of NAbs relative to species differences, immune function, and ecology (27, 28, 30, 93).

Many studies have shown species and breed effects on levels of NAbs (6, 27, 28, 40, 42). However, to our knowledge, antigen specificity of NAbs has not been considered in comparative studies. Perhaps the best cited example of using NAbs as a comparative trait is Matson et al. (122). In that study, the authors compared levels of NAbs using agglutination assays with rabbit and trout red blood cells. Although the average NAb levels in the two assays were correlated (R^2^ = 0.62), there were cases where species effects were not identical for anti-rabbit and anti-trout RBC immunoglobulins. For example, the Chilean pintail (*Anas georgica spinicauda*) and white-winged wood duck (*Cairina scutulata*) had similar titers of NAbs binding rabbit red blood cells but dissimilar titers of NAbs binding trout red blood cells. In contrast, South Georgia pintail (*Anas georgica georgica*) and black-bellied tree duck (*Dendrocygna autumnalis*) had the opposite pattern (122). Other studies have reported variation in NAbs among chicken breeds (22, 55), but breed effects are not identical among NAb antigens (40). These inconsistencies among antigen-specific NAbs may reflect complexity in the genetic control of natural antibody diversity (62–65, 114).

Natural antibodies are considered important to innate immune defense (9, 11, 13), but it remains unclear if antigen specificity of NAbs affects defense. Monoclonal NAbs do not bind uniformly to all pathogens (8, 24) and they do not neutralize different pathogens equally *in vitro* (24). This suggests that antigen specificity could affect defenses provided by NAbs. Animal studies with chickens and pigs have reported positive (41, 123) or absent (1, 56) relationships between antigen-specific NAbs and survival. In pigeons, Owen et al. (21) observed that some antigen-specific NAbs were predictive of bird resistance to internal and external parasites, but other antigen-specific NAbs were not. In addition, the authors showed that pigeons with a more diverse repertoire of antigen-specific NAbs were more resistant to parasites (21). These various studies suggest that antigen-specific NAbs provide defense against a restricted range of parasites or pathogens, reinforcing the idea that immune defense from NAbs relies on a diverse repertoire of these immunoglobulins (9, 84).

The mechanism(s) of defense by NAbs are not entirely understood and remain an active area of investigation (1–6, 11, 14–17, 54, 100, 106, 107). Although NAbs are germline-encoded and produced prior to infection, evidence suggests that production of NAbs can increase following immune stimulation (52, 54, 68, 106, 106, 110, 111). However, increased production of NAbs appears to be antigen specific. Some antigen-specific NAbs increase with immune challenge, but others remain unchanged (27, 52, 54, 106, 112, 113). In addition, relationships between NAbs, adaptive antibodies, and other immune effectors vary depending on antigen specificity (49, 83). Matson et al. (122) measured 13 immunological parameters that included NAbs, complement proteins, antimicrobial activity, and leukocyte counts from 10 species of waterfowl. The authors used principal component analysis as a statistical approach to group correlated variables. The NAb and complement levels were measured using the hemagglutination-hemolysis assay that combines a measure of complement protein activity (blood cell lysis) with NAbs that mediate the binding of complement to the target blood cells (i.e., classical complement pathway) (21, 24, 77). As discussed above, Matson et al. (122) used trout and rabbit red blood cells in separate assays of the same samples. The lysis (complement) and antimicrobial measures grouped into different PCs based on antigen. One PC showed a positive correlation with trout cell lysis and killing of *Staphylococcus aureus* bacteria. In contrast, a different PC correlated positively with rabbit cell lysis but negatively with the killing of *S. aureus*. In this example, relationships between NAbs, complement, and bacteria killing changed depending on NAb antigen specificity (100). These studies strongly suggest that antigen-specificity of NAbs affects interactions with pathogens and the defenses provided by these immunoglobulins. Accurate and comprehensive understanding of NAbs and immune function requires consideration of antigen specificity.

The data reported here reveal that natural antibodies in poultry are composed of a diverse repertoire of immunoglobulins. Our data align with previous studies of NAbs in chickens that show both breed and selection line affect natural antibody levels (6, 22, 40, 42, 47–49, 52, 55). Selective breeding of poultry has revealed that some antigen-specific NAbs are associated with pathogen resistance (39–41) and survival (1, 56). However, the mechanism(s) of these effects are unknown. Natural antibodies may provide important immune defenses in chickens or serve as relevant markers for selection. In either case, identifying the effects of antigen specificity could reveal more targeted strategies for improving traits in these important livestock.

## 5 Conclusions

Natural antibodies are important components of the vertebrate immune system, and these molecules have proven valuable in studies of immune function, ecology, evolution, and livestock selection. These molecules are defined as polyreactive with low specificity (11, 13), but evidence from our studies and the literature reveal that antigen-specific NAbs are independent and have unique interactions with pathogens and parasites (8, 24). Thus, antigen-specific NAbs are not interchangeable. A comprehensive understanding of NAbs requires consideration for functional differences between antigen-specific NAbs and characterization of immunoglobulin diversity in the NAb repertoire. This leads to the question of how researchers should proceed with measurements of NAbs? Natural antibodies should be appreciated as a group of diverse immunoglobulins rather than treated as a singular effector (9, 84). Going forward, we recommend three approaches to the study of NAbs. The first is to reconcile the similarities and differences among antigen-specific NAbs (114). For example, by combining western blot analyses and affinity chromatography, researchers may be able to identify how antigen-specific NAbs are related (46, 51, 124, 125). Second, researchers could use a panel of antigens to measure the NAb repertoire (21), or design ELISAs that utilize several antigens at once (e.g., multiplex ELISA) (21, 126–128). These approaches would yield more complete measures of the NAb repertoire. Finally, as biochemical and genetic resources become available for non-model animal species, researchers should endeavor to determine the cellular and molecular characteristics of NAbs in different animal species.

## Data availability statement

The raw data supporting the conclusions of this article will be made available by the authors, without undue reservation.

## Ethics statement

The animal study was approved by WSU Institutional Animal Care and Use Committee. The study was conducted in accordance with the local legislation and institutional requirements.

## Author contributions

KW: Data curation, Formal analysis, Investigation, Visualization, Writing – original draft, Writing – review & editing. JF: Resources, Writing – review & editing. JO: Conceptualization, Formal analysis, Funding acquisition, Investigation, Methodology, Project administration, Supervision, Validation, Visualization, Writing – review & editing.
